# A nationwide study of the risks of major mental disorders among the offspring of parents with rheumatoid arthritis

**DOI:** 10.1038/s41598-022-08834-5

**Published:** 2022-03-23

**Authors:** Hsien-Jane Chiu, Cheuk-Kwan Sun, Shih-Jen Tsai, Ya-Mei Bai, Kuo-Chuan Hung, Ju-Wei Hsu, Kai-Lin Huang, Tung-Ping Su, Tzeng-Ji Chen, Andrew Sun, Yu-Shian Cheng, Mu-Hong Chen

**Affiliations:** 1 Department of Psychiatry, Taoyuan Psychiatric Center, Ministry of Health and Welfare, Taoyuan, Taiwan (R.O.C.); 2 Institute of Hospital and Health Care Administration, National Yang Ming Chiao Tung University, Taipei, Taiwan; 3 Department of Emergency Medicine, E-Da Hospital, Kaohsiung City, Taiwan (R.O.C.); 4 School of Medicine for International Students, I-Shou University, Kaohsiung City, Taiwan (R.O.C.); 5 Department of Psychiatry, Taipei Veterans General Hospital, No. 201, Shih-Pai Road, Sec. 2, Taipei, 11217 Taiwan (R.O.C.); 6 Division of Psychiatry, School of Medicine, National Yang-Ming University, Taipei, Taiwan (R.O.C.); 7 Department of Anesthesiology, Chi Mei Medical Center, Tainan, Taiwan (R.O.C.); 8 Department of Psychiatry, General Cheng Hsin Hospital, Taipei, Taiwan (R.O.C.); 9 Department of Family Medicine, Taipei Veterans General Hospital, Taipei, Taiwan (R.O.C.); 10 Emory College of Arts and Sciences, Emory University, Atlanta, GA USA; 11 Department of Psychiatry, Tsyr-Huey Mental Hospital, Kaohsiung Jen-Ai’s Home, No. 509, Fengping 1st Rd., Daliao Dist., Kaohsiung City, Taiwan (R.O.C.); 12 Institute of Biomedical Sciences, National Sun Yat-Sen University, Kaohsiung City, Taiwan (R.O.C.)

**Keywords:** Health care, Medical research, Rheumatology, Risk factors

## Abstract

Rheumatoid arthritis (RA) may share genomic risks with certain mental disorders. This study aimed at investigating associations between parental RA and risks of mental disorders in offspring. Using the National Health Insurance Research Database (2001–2010), we conducted a matched cohort study involving two parent–child cohorts (i.e., RA-parent–child cohort and non-RA-parent–child cohort) between which risks of major mental disorders in offspring were compared. There were 23,981 parent–child pairs in the RA-parent–child cohort and 239,810 in the non-RA-parent–child cohort. Preliminary analysis demonstrated increased risks of autism spectrum disorders (ASDs) [Odds ratio (OR) 1.47; 95% confidence interval (CI) 1.05–2.07], attention-deficit/hyperactivity disorder (ADHD) [OR 1.34; (95% CI 1.17–1.54)], bipolar disorder [OR 1.41 (95% CI 1.17–1.70)], and major depressive disorder [OR 1.20 (95% CI 1.07–1.35)] associated with parental RA. Sub-group analysis further showed higher risks of the four disorders in children of mothers with RA but not those from fathers with RA. Higher risks of ASDs and ADHD were not noted in children of mothers with RA before childbirth. Maternal RA, but not paternal RA or mothers diagnosed with RA before childbirth, was associated with increased risks of multiple mental disorders in their offspring, suggesting potential contributions of maternal genetic factors to ASDs and ADHD development in offspring.

## Introduction

Rheumatoid arthritis (RA), a chronic inflammatory autoimmune disease that affects between 0.5% and 1% of the global population, is highly inheritable^1^. A previous nationwide cohort study has revealed a notably increased risk of RA in offspring of mothers with the disease^2^, highlighting a genetic predisposition to the disease. On the other hand, another large-scale study has demonstrated a positive association of the genetic susceptibility to RA with cognitive and mental symptoms in adolescents and children, suggesting that RA and mental disorders may have shared genomic risks^3^. Such a link between two apparently distinct categories of immune and mental diseases^3^ has stimulated a number of studies investigating whether parental RA would predispose their offspring to the development of mental phenotypes. Consistently, previous studies have identified a positive association between parental RA and the risk for autism spectrum disorders (ASDs)^4,5^. Some authors also demonstrated an increased incidence of attention-deficit/hyperactivity disorder (ADHD) in offspring of mothers with RA^6^. Possible mechanisms include shared genetic vulnerability between mental disorders and RA^3^ as well as the possibility that certain conditions during pregnancy of RA mother may increase the risks of mental disorders in offspring^7^.

On the other hand, affective and mood disturbances, which are not uncommon among patients with RA, are mostly regarded as comorbidities of RA secondary to chronic pain, fatigue, and physical disability^8^. Indeed, a Danish nationwide cohort study failed to show any significant association between affective disorders in offspring and maternal RA^2^. Nevertheless, that study did not discuss specific forms of affective disorders (e.g., depression, bipolar disorder). Although previous studies also revealed an inverse relationship between RA and Schizophrenia^9,10^, there was no large-scale study addressing the issue.

Therefore, the present nationwide cohort study aimed at investigating the association of parental RA with five mental disorders, namely ASDs, ADHD, major depressive disorder (MDD), bipolar disorder, and Schizophrenia in offspring as well as the differential impact of parental sex by comparing the risk of disease in offspring between the paternal and maternal lines. To elucidate whether the possible associations were due to genetic vulnerability or pregnancy-related conditions, the odd ratios of mental disorders were compared between offspring from mothers with and without an established diagnosis of RA before childbirth.

## Methods

### Data source

This study used data from Taiwan's National Health Insurance (NHI), which is a mandatory universal health insurance program that was implemented in 1995 to offer comprehensive medical care coverage for all Taiwanese residents. The National Health Research Institute (NHRI) is in charge of the entire insurance claims database, namely the National Health Insurance Research Database (NHIRD), which consists of healthcare data from > 99% of the entire Taiwan population. The NHRI audits and releases the NHIRD for research purposes. Claims data of subjects included in the NHIRD are anonymous to protect patient confidentiality. Comprehensive information on insured subjects is included in the database, including demographic data, dates of clinical visits, and diagnoses. All of the information was linked to a unique personal identification number assigned to each resident in Taiwan. The recorded family kinships in the NHIRD were used for genealogy reconstruction following the methods previously reported^11,12^. The diagnostic codes used were based on the International Classification of Diseases, 9th Revision, Clinical Modification (ICD-9-CM). The NHIRD has been extensively used in many epidemiologic studies in Taiwan^11–14^. The Institutional Review Board of Taipei Veterans General Hospital reviewed the protocol of the current study and approved the waiving of informed consent in view of the retrospective design (Approval No. IRB/REC 2018-07-016AC). This study was performed in accordance with the Declaration of Helsinki.

### Inclusion criteria of parents with RA and their offspring

The present study adopted two parent–child cohorts (i.e., the RA parent–child cohort and the non-RA-parent–child cohort). Following the identification of parental RA, we traced the occurrence of five mental disorders, namely ADHD, ASDs, schizophrenia, bipolar disorder, and MDD, in their offspring to generate the RA parent–child cohort by including all parents diagnosed with RA as well as their offspring within the study period between 2001 and 2010 from the NHIRD database. Then we used age- and gender-matched non-RA parent–child pairs as controls for comparison in a ratio of 1:10. For instance, a 42-year-old mother with RA having a 12-year-old daughter would be matched with ten 42-year-old mothers without RA having a 12-year-old daughter. In addition, to avoid duplicated selection, parents with more than one child were excluded from the study. To differentiate between genotypic and pregnancy condition-related impacts of maternal RA on the risks of developing the five mental disorders in offspring, we first identified women with concomitant diagnoses of pregnancy and RA whose offspring were compared with those from mothers without diagnosis of RA (in a ratio of 1 to 10) with comparable follow-up periods.

### Disease classification and the assessment of covariates

All of the diagnoses in the current study were drawn from the NHIRD database. In Taiwan, the diagnostic information of severe systemic autoimmune diseases, including RA, is sent to the insurance administration for review by commissioned expert panels to confirm the diagnosis based on the updated classification criteria of RA (ICD-9-CM code: 714.0)^15^. All the information of patients with severe systemic autoimmune diseases is included in the database of Catastrophic Illness^16^ based on which patients are entitled to waive the medical co-payment after confirmation of the diagnosis. The diagnoses of major mental disorders including schizophrenia (ICD-9-CM code: 295), bipolar disorder (ICD-9-CM codes: 296, except for 296.2, 296.3, 296.9, and 296.82), major depressive disorder (ICD-9-CM codes: 296.2 and 296.3), ASDs (ICD-9-CM code: 299), and ADHD (ICD-9-CM code: 314) were made by board-certified psychiatrists based on their clinical judgment and diagnostic interviews from at least two clinical visits. Patients who were diagnosed only once were excluded from the current analysis. With regard to the diagnosis of schizophrenia, bipolar I disorder, and major depressive disorder, the final diagnosis was confirmed based on the diagnostic hierarchy of the three disorders. As for ADHD and ASDs, each diagnosis was considered a separate disorder in the current study. Demographic data, including age, sex, place of residence, and income status in 2010, are displayed in Table 1 and were considered to be potential confounding factors. The place of residence was classified into five categories according to the level of urbanization^17^. Because the age of offspring was matched in the current study, the lengths of follow-up were identical between the groups for comparison.

**Table 1:** Demographic characteristics and prevalence of mental disorders among the offspring of parents with and without rheumatoid arthritis.

	Parents with RA and their children (n = 23,981)	Parents without RA and their children (n = 239,810)	*p*-value
Age of parents (years, SD)	61.66 (12.93)	61.66 (12.93)	0.999
**Sex of parents (n, %)**			1.000
Father	5281 (22.0)	52,810 (22.0)	
Mother	18,700 (78.0)	187,000 (78.0)	
Age of children (years, SD)	33.95 (13.14)	33.95 (13.15)	0.948
**Sex of children (n, %)**			0.945
Son	14,381 (60.0)	143,754 (59.9)	
Daughter	9600 (40.0)	96,056 (40.1)	
**Mental disorders of parents (n, %)**			
Schizophrenia	99 (0.4)	1276 (0.5)	**0.016**
Bipolar disorder	134 (0.6)	981 (0.4)	**0.001**
Major depressive disorder	718 (3.0)	4834 (2.0)	** < 0.001**
RA of offspring (n, %)	84 (0.4)	174 (0.1)	** < 0.001**
**Mental disorders of children (n, %)**			
ADHD	241 (1.0)	1819 (0.8)	** < 0.001**
ASD	38 (0.2)	259 (0.1)	**0.030**
Schizophrenia	172 (0.7)	1838 (0.8)	0.435
Bipolar disorder	128 (0.5)	908 (0.4)	** < 0.001**
Major depressive disorder	310 (1.3)	2559 (1.1)	**0.001**
**Level of urbanization (n, %)**			0.167
1 (most urbanized)	6439 (26.9)	63,612 (26.5)	
2	8021 (33.4)	81,350 (33.9)	
3	3130 (13.1)	31,459 (13.1)	
4	2158 (9.0)	22,162 (9.2)	
5 (most rural)	4233 (17.7)	41,227 (17.2)	
**Income-related insured amount**			0.224
≤ 15,840 NTD/month	13,079 (54.5)	130,038 (54.2)	
15,841–25,000 NTD/month	5821 (24.3)	57,807 (24.1)	
≥ 25,001 NTD/month	5081 (21.2)	51,965 (21.7)	

Note: bold figures indicate statistical significance.

*Abbreviations: RA* rheumatoid arthritis, *SD* standard deviation, *ADHD* attention deficit hyperactivity disorder, *ASD* autism spectrum disorder, *NTD* New Taiwan dollar.

### Statistical analysis

We performed Chi-square tests and F-tests for categorical and continuous variables, respectively, to identify the possible confounders of the study outcomes. Potential confounders were then adjusted with logistic regression before subsequent analysis. Four logistic regression models were adopted to investigate the offspring risks of major mental disorders associated with parental RA; Model 1 involved the adjustment for demographic data, including the levels of urbanization and income, which are considered potential confounders because of their associations with health care utilization as well as the prevalence of parental mental disorders and RA in offspring^18^. Model 2 additionally adjusted for the prevalence of RA in offspring and that of parental mental disorders, including schizophrenia, bipolar disorder, and major depressive disorder. We did not adjust for parental ADHD (n = 56, 0.02%) and ASDs (n = 7, 0.003%) in the regression model because of the very low prevalence. We further performed separate analyses on offspring of mothers with RA (Model 3) and those of fathers with RA (Model 4). Finally, although RA is most common among those aged 40–70 with an increasing incidence with age^19^, the association between intrauterine exposure to maternal RA and the risk of developing ADHD and ASDs in childhood remains unclear. To address this issue, we selected mothers with RA before childbirth and age-/sex-matched in a ratio of 1:10 with those without RA and compared the risks of ADHD and ASDs in their children using logistical regression with adjustment of demographic data. Only risks of ADHD and ASDs were calculated in relation to the analysis of RA before or after childbirth because the sample sizes of the other two disorders were too small to conduct the analysis. A two-tailed *p*-value of less than 0.05 was considered statistically significant. All data processing and statistical analyses were performed with Statistical Package for Social Science (SPSS) version 17 (Chicago, SPSS Inc.) and Statistical Analysis Software (SAS) version 9.1 (SAS Institute, Cary, NC).

### Ethics approval and consent to participate

Taipei Veterans General Hospital Institutional Review Board approved the protocol and procedures of the current study (Approval No. 2018-07-016AC).

## Results

### Associations of parental rheumatoid arthritis with mental disorders in offspring

Table 1 shows the demographic characteristics (e.g., age, sex, income, and urbanization) and prevalence of mental disorders among the parents and the offspring of parents with or without RA. Our results showed no coincidence of RA in both parents. Comparing 23,981 parent–child pairs in the RA-parent–child cohort with 239,810 parent–child pairs in the non-RA-parent–child cohort demonstrated no significant differences in income and urbanization between the two cohorts. However, preliminary analysis showed significantly higher prevalence of ASDs (*p* < 0.03), ADHD (*p* < 0.001), bipolar disorder (*p* < 0.001) and major depressive disorder (*p* = 0.001) in offspring of parents with RA compared with that in offspring of parents without RA but such an association was not observed between parental RA and schizophrenia in offspring (*p* = 0.435).

### Identification of risks of mental disorders in offspring of parents with rheumatoid arthritis after adjustments for confounders

After adjustments for the demographic characteristics (Model 1) as well as the prevalence of RA in offspring and parents with mental disorders (Model 2), logistic regression analysis on the correlation between parental RA and mental disorders of their children revealed significantly higher risks of ASDs [Odds ratio (OR) 1.47 and 95% confidence interval (CI) 1.05–2.07], bipolar disorder [OR 1.41; 95% CI 1.17–1.70], ADHD [OR 1.34; 95% CI 1.17–1.54], and major depressive disorder [OR 1.20; 95% CI 1.07–1.35] in offspring of parents with RA despite the lack of notable association for schizophrenia [OR 0.96; 95% CI 0.82–1.13] (Table 2). Further analysis on the risks of mental disorders in offspring with only maternal RA (Model 3) and those with only paternal RA (Model 4) showed that only offspring of mothers with RA had significantly higher risks of ASDs [OR 1.49; 95% CI 1.01–2.20], bipolar disorder [OR 1.47; 95% CI 1.20–1.81], ADHD [OR 1.37; 95% CI 1.17–1.60], and major depressive disorder [OR 1.20; 95% CI 1.05–1.37] but not that of schizophrenia [0.96; 95% CI 0.80–1.15]. Children of fathers with RA did not have higher risks of any mental disorders included in the present study (Table 2).

**Table 2:** Logistic regression on associations between parental rheumatoid arthritis and mental disorders of their children.

	Children mental disorders (OR, 95% CI)				
	ADHD	ASD	Schizophrenia	Bipolar disorder	Major depressive disorder
**Model 1**					
Parental RA					
Presence (n = 23,981)	**1.36 (1.18–1.56)**	**1.49 (1.06–2.10)**	0.96 (0.82–1.13)	**1.43 (1.19–1.72)**	**1.22 (1.09–1.28)**
Absence (n = 239,810)	1 (Ref)	1 (Ref)	1 (Ref)	1 (Ref)	1 (Ref)
**Model 2**					
Parental RA					
Presence (n = 23,981)	**1.34 (1.17–1.54)**	**1.47 (1.05–2.07)**	0.96 (0.82–1.13)	**1.41 (1.17–1.70)**	**1.20 (1.07–1.35)**
Absence (n = 239,810)	1 (Ref)	1 (Ref)	1 (Ref)	1 (Ref)	1 (Ref)
**Model 3**					
Paternal RA					
Presence (n = 5281)	1.26 (0.93–1.70)	1.42 (0.70–2.87)	0.96 (0.66–1.28)	1.17 (0.77–1.79)	1.20 (0.92–1.57)
Absence (n = 52,810)	1 (Ref)	1 (Ref)	1 (Ref)	1 (Ref)	1 (Ref)
**Model 4**					
Maternal RA					
Presence (n = 18,700)	**1.37 (1.17–1.60)**	**1.49 (1.01–2.20)**	0.96 (0.80–1.15)	**1.47 (1.20–1.81)**	**1.20 (1.05–1.37)**
Absence (n = 187,000)	1 (Ref)	1 (Ref)	1 (Ref)	1 (Ref)	1 (Ref)

Model 1: data from both parents, adjusted for demographic data (income, residence);

Model 2: data from both parents, adjusted for demographic data, prevalence of parental mental disorders and offspring RA.

Model 3: data from only fathers, adjusted for demographic data, prevalence of parental mental disorders and offspring RA.

Model 4: data from only mothers, adjusted for demographic data, prevalence of parental mental disorders and offspring RA.

Note: bold figures indicate statistical significance.

*Abbreviations: RA* rheumatoid arthritis, *OR* odds ratio, *CI* confidence interval, *ADHD* attention-deficit/hyperactivity disorder*, ASDs* autism spectrum disorders, *Ref* reference.

### Assessment of the impact of pregnancy on risks of mental disorders in offspring

To investigate the potential influences of pregnancy condition on the development of mental disorders in offspring, additional analysis was performed focusing on mothers with RA before childbirth. Table 3 summarizes the demographic data and risks of ADHD or ASDs among the offspring of mothers with and without RA before childbirth. There were no significant differences in demographic characteristics between the RA-mother–child cohort and the non-RA-mother–child cohort. There were also no significant differences in the risks of ADHD or ASDs between offspring of mothers with RA before childbirth and those of mothers without RA before childbirth.

**Table 3:** Risks of attention-deficit/hyperactivity disorder and autism spectrum disorders among offspring of mothers with rheumatoid arthritis diagnosed before childbirth.

	Mothers with RA before childbirth and their children (n = 324)	Mothers without RA before childbirth and their children (n = 3240)	*p*-value	OR, 95% CI*
Age of parents (years, SD)	38.53 (5.25)	38.56 (5.21)	0.912	
Age of children (years, SD)	7.89 (4.24)	8.38 (4.40)	0.059	
**Sex of children (n, %)**			0.221	
Son	162 (50.0)	1736 (53.6)		
Daughter	162 (50.0)	1504 (46.4)		
**Maternal mental disorders (n, %)**				
Schizophrenia	0 (0)	10 (0.3)	0.614	
Bipolar disorder	5 (1.5)	10 (0.3)	**0.008**	
Major depressive disorder	3 (0.9)	47 (1.5)	0.621	
**Level of urbanization (n, %)**			0.809	
1 (most urbanized)	83 (25.6)	796 (24.6)		
2	126 (38.9)	1184 (36.5)		
3	37 (11.4)	428 (13.2)		
4	35 (10.8)	377 (11.6)		
5 (most rural)	43 (13.3)	455 (14.0)		
**Income-related insured amount**			0.358	
≤ 15,840 NTD/month	50 (15.4)	518 (16.0)		
15,841–25,000 NTD/month	136 (42.0)	1472 (45.4)		
≥ 25,001 NTD/month	138 (42.6)	1250 (38.6)		
RA of offspring (n, %)	0 (0)	0 (0)		
**Mental disorders of children (n, %)**				
ADHD (n, %)	17 (5.2)	126 (3.9)	0.234	1.47 (0.86–2.52)
ASDs (n, %)	2 (0.6)	21 (0.6)	> 0.999	1.02 (0.24–4.42)

Note: bold figures indicate statistical significance.

*Abbreviations: RA* rheumatoid arthritis, *OR* odds ratio, *CI* confidence interval, *SD* standard deviation, *NTD* new Taiwan dollar, *ADHD* attention-deficit/hyperactivity disorder*, ASDs* autism spectrum disorders.

*Adjusted for demographic data, offspring RA, and maternal mental disorders.

## Discussion

To the best of our knowledge, this is the first nationwide population-based survey studying the risks of developing specific mental disorders in the offspring of parents with rheumatoid arthritis. Review of literature identified only one Asian study investigating the impact of maternal RA on the risk of ASDs in newborn children^20^, while the rest of the studies were from European countries^4–6^. Regarding the parental influence of RA on the risk of ADHD in the offspring, there was one nationwide study addressing this issue^6^ and two other reports assessing the influence of genetic risks^3^ and familial history^21^ on disease development, respectively. When focused on the risks of affective disorder and schizophrenia in the offspring of parents with RA, an investigation into the associations did not specifically address different types of affective disorders (e.g., depression or bipolar disorders)^2^.

Utilizing a nationwide database, our study revealed significant associations of maternal RA with ASDs, ADHD as well as major depressive and bipolar disorders. Importantly, these associations remained significant after adjustments for confounding factors including income and level of urbanization as well as the prevalence of parental mental disorders and RA in offspring (Table 2). Our results showed that maternal RA was associated with increased risks of ASDs, bipolar disorder, ADHD, and MDD by about 50%, 50%, 40%, and 20%, respectively, without a significant impact on the risk of schizophrenia.

Of the five mental disorders included in the present study, most previous reports focused on ASDs which were also shown to have the highest association with parental RA in our investigation. Our result was consistent with that of a previous meta-analysis^4^ demonstrating a similar increase in risk of ASDs in offspring with maternal RA [odds ratio (OR) 1.39; 95% CI 1.16–1.67]. The difference was that the previous meta-analysis only assessed the impact of maternal RA. Moreover, with the exception of one Taiwanese report^20^, all other studies in that meta-analysis were from western countries^4^. Interestingly, the previous Taiwanese study failed to show a significant association between maternal RA and ASDs in offspring despite its demonstration of a similar increase in relative risk (RR: 1.42; 95% CI 0.60–3.40) with a study period (from 2001 to 2012) comparable to that of the present investigation (from 2001 to 2010)^20^.

One issue to be addressed is whether the risk of developing ASDs in offspring is genetic or pregnancy-related because previous studies have shown that certain immune mediators may reach fetal circulation or penetrate placental and blood–brain barriers during pregnancy^7,22–24^. Because the age of RA onset is highest among adults in their sixties well after the fertile period^25^, the previous study including only children born to mothers with an established diagnosis of RA^20^ may focus more on the pregnancy-related influence rather than the genetic impact on the development of ASDs in offspring because the majority of the RA population (e.g., aged) was not investigated. Therefore, the lack of a significant correlation between maternal RA and offspring ASDs in that study with a relatively small sample size (n = 673)^20^ may either suggest a non-significant impact of pregnancy on the development of ASDs in offspring or that the sample size was not large enough to achieve statistically significant results. In contrast, although the present study also showed a non-significant association between ASDs in offspring and maternal RA when only the mothers with RA before childbirth were considered, the risk of ASDs in offspring was significantly higher after including the entire data from maternal side (Table 2). Taking into account the large sample size (n = 18,700) and the inclusion of mothers of all ages in the current study, our findings could better reflect the maternal genetic impact on psychiatric disorder development in offspring. Our results support that ASDs in offspring were positively related to maternal RA genetically but the association between pregnancy-related conditions and RA remained inconclusive. On the other hand, our study demonstrated no significant association between the risk of ASDs in offspring and paternal RA, implicating a stronger maternal influence. This is supported by the findings of common genetic pathways between autoimmune disease and ASDs as well as the identification of several susceptibility alleles for autoimmune diseases in both the offspring with ASDs and their mothers^26–28^.

Similarly, our results regarding ADHD also implied that the genetic association on the maternal side may be a possible explanation for an elevated risk of ADHD in offspring of parents with RA, which was also found only in offspring of mothers but not fathers with RA and not in those whose mothers had a diagnosis of RA before childbirth. The findings were consistent with those of a previous study that also reported a higher risk of ADHD in offspring of mothers with RA^6^. Moreover, the genetic association between ADHD and autoimmune disease has been found in genetic and familial studies of ADHD^3,21,29^. Similar to ASDs, the absence of a significant correlation between maternal RA diagnosed before childbirth and ADHD in offspring does not support the hypothesis of pregnancy-related influence through transplacental transfer of immune mediators. Overall, our results suggest that a genetic association especially on the maternal side, instead of the condition of pregnancy, may contribute to an elevated risk of the two neurodevelopmental disorders of ADHD and ASDs. Nevertheless, more studies are needed to elucidate our findings.

In addition, our study showed a significant positive association between bipolar disorder in offspring and maternal RA. There was only one related study that demonstrated no significant association between affective disorders in offspring and maternal RA without providing detailed information about the definition of affective disorders^2^. In contrast, a previous meta-analysis that reported an elevated risk of bipolar disorder in patients with RA (OR 2.06 [1.34–3.17]) may suggest a link between bipolar disorder and RA^30^. Consistently, previous studies have shed some light on the possible association between the two disorders by demonstrating the involvement of neuro-inflammation in the pathogenesis of bipolar disorder^7^, which has been shown to be associated with certain peripheral inflammatory markers^31^.

On the other hand, although it is well-documented that depression is highly prevalent in patients with RA and related to poorer RA outcomes^32^, the association between parental RA and depression in offspring has not been reported. The current study, which is the first to address this issue, revealed increased odds of major depressive disorder in offspring of mothers diagnosed with RA. Again, a previous study has reported that pro-inflammatory cytokines and neuro-inflammation have a part to play in the brain pathology of depression^33^. Therefore, it is possible that there may be a common genetic pathway or pregnancy-related condition between affective disorder in offspring and parental RA. Further investigations are warranted to support the finding.

The association between RA and Schizophrenia has also aroused much interest^9^. Although previous reports have demonstrated a significant inverse relationship between the two diseases^9,10^, the present study showed neither significant positive nor negative association between parental RA and schizophrenia in offspring.

### Strengths and limitations

The present study represented a large-scale nationwide population-based study that covered most of the Taiwanese population (sample size = 263,791 from 2001 to 2010). Since most people are covered by NHI in Taiwan, we were able to collect reliable demographic data and adjust our results accordingly. The current study investigated multiple mental disorders, including those that were not well-addressed in previous studies. Besides, because most previous reports were from western countries, the present investigation provided related data from Asia. Furthermore, in contrast to previous studies, one of the merits of the present investigation was to differentiate between the paternal and maternal genetic impact as well as between maternal genetic and pregnancy-related influence on the risks of psychiatric disorders in offspring.

Nevertheless, the current study had its limitations. First, despite the large database, the size of the targeted population (e.g., ASDs) may still be too small to conduct a large-scale comparison. Second, because laboratory data were not accessible, the accuracy of physician-based diagnosis of RA remained unconfirmed. Similarly, although the diagnosis of mental diseases was based on physicians’ judgment and patient’s history, none of them were available for validation. On the other hand, several studies have shown a high validity of diagnosis codes from NHIRD data^16^. In addition, as a national healthcare policy, information on the diagnosis of RA made by physicians is sent to the insurance administration for a strict review by commissioned expert panels for confirmation based on the updated classification criteria of RA^15^ to ensure diagnostic accuracy. Third, information regarding disease severity, sero-status regarding RA biomarkers, and response to medical treatment as well as pregnancy and other maternal health conditions, which may be related to the development of mental disorders in offspring^34–36^, was not available. Therefore, we cannot rule out the influences of these potential confounding factors on our study outcomes. With the growing linkages among different databases, future studies based on NHIRD data may be able to overcome these shortcomings^16^. Fourth, by using the diagnostic codes from NHIRD data, the risk of selection bias may be an issue in the current study as we only included participants who sought medical attention because of relatively severe RA. Further studies are warranted to assess the potential impact of the severity of RA on the risk of mental disorders. Fifth, due to the small numbers of patients diagnosed with mental disorders identified in the current study, the absolute risks would be too small to be significant from a public health perspective. Therefore, the present study only focused on the relative risk [i.e., odds ratio (OR)]. Nevertheless, this investigation would open up avenues for further research through underscoring the potential genetic link between parental RA and the development of psychiatric disorders in offspring. Sixth, although the identification of maternal RA and increased risks of ASDs and ADHD in offspring may suggest a causal relationship, further studies are warranted to support our findings. Finally, the results from studying a specific ethnic group limit their application in populations of other ethnic origins. Following the same path, further studies from other countries may elucidate the possible effects of ethnicity on the associations.

## Conclusions

Through differentiating between paternal and maternal genetic influences as well as between maternal genetic and pregnancy-related impacts on the risks of five psychiatric disorders in offspring, the current nationwide population-based cohort study demonstrated a positive association of maternal rheumatoid arthritis (RA) (but not paternal RA or the subgroup of mothers with diagnosis of RA before childbirth) with four out of the five investigated mental disorders in offspring (i.e., ASDs, ADHD, bipolar disorder, and MDD). Further large-scale cohort studies are warranted to support our findings.

## Data Availability

The data underlying this article will be shared on reasonable request to the corresponding author.

## Abbreviations

RA: Rheumatoid arthritis
ASDs: Autism spectrum disorders
ADHD: Attention-deficit/hyperactivity disorder
MDD: Major depressive disorder
NHI: Taiwan's National Health Insurance
NHRI: National Health Research Institute
NHIRD: National Health Insurance Research Database
ICD-9-CM: International Classification of Diseases, 9th Revision, Clinical Modification
OR: Odds ratio
CI: Confidence interval
RR: Relative risk
